# Adherence to the Mediterranean Diet is Associated with Better Sleep Quality in Italian Adults

**DOI:** 10.3390/nu11050976

**Published:** 2019-04-28

**Authors:** Justyna Godos, Raffaele Ferri, Filippo Caraci, Filomena Irene Ilaria Cosentino, Sabrina Castellano, Fabio Galvano, Giuseppe Grosso

**Affiliations:** 1 Department of Biomedical and Biotechnological Sciences, University of Catania, 95123 Catania, Italy; justyna.godos@student.uj.edu.pl (J.G.); fgalvano@unict.it (F.G.); 2 Oasi Research Institute - IRCCS, 94018 Troina, Italy; rferri@oasi.en.it (R.F.); carafil@hotmail.com (F.C.); fcosentino@oasi.en.it (F.I.I.C.); 3 Department of Drug Sciences, University of Catania, 95125 Catania, Italy; 4 Department of Educational Sciences, University of Catania, 95124 Catania, Italy; sabrinacastellano@hotmail.it

**Keywords:** Mediterranean diet, sleep quality, cognitive decline, dementia, weight status, mental health, obesity, cohort, Italy

## Abstract

Background: Sleep quality has been associated with human health and diseases, including cognitive decline and dementia; however major determinants of sleep disorders are largely unknown. The aim of this study was to evaluate the association between sleep quality and adherence to the Mediterranean dietary pattern in a sample of Italian adults. Methods: A total of 1936 individuals were recruited in the urban area of Catania during 2014–2015 through random sampling. A food frequency questionnaire and validated instruments were used to assess the adherence to the Mediterranean diet and sleep quality (Pittsburg sleep quality index). Multivariate logistic regressions were performed to determine the association between exposure and outcome. Results: A total of 1314 individuals (67.9% of the cohort) reported adequate sleep quality: for each point increase of the Mediterranean diet score, individuals were 10% more likely to have adequate sleep quality. In an additional analysis stratifying the sample by weight status, the association between sleep quality and high adherence to the Mediterranean diet was observed only among normal/overweight individuals but not in obese participants. Conclusions: high adherence to a Mediterranean diet is associated with better sleep quality either toward direct effect on health or indirect effects through improvement of weight status.

## 1. Introduction

Epidemiological evidence suggests that sleeping habits might be related to human health, including cardio-metabolic and mental health outcomes [1,2]. Most of existing evidence focuses on sleep duration, suggesting that lack of sleep may exert negative effects on a variety of systems [3]. The mechanisms mediating the relation between sleep and health status are not entirely clear, but are likely to be multifactorial, involving hormonal disruption, metabolic impairment, and inflammatory processes [4,5]. Although short term sleep deprivation is associated to decrements in the psychomotor vigilance task, the most consistent finding animal studies showed that chronic unhealthy sleeping behaviors may impact central nervous system structural plasticity in different ways, including reduction of spine density and attenuation of synaptic efficacy in the hippocampus [6]. Long-term changes in sleep quality and architecture have been related to cognitive impairment; while the incidence of sleep disorders may increase with normal aging, further impairment of sleep-dependent memory consolidation has been observed in relation with neurodegenerative diseases, including dementias and Alzheimer’s disease [7]. It is not clear whether sleep disturbances occur with higher rate in individuals having cognitive impairment or dementia, or they may also represent an independent risk factor for such pathological conditions. However, sleep disorders and cognitive decline seem to be somehow connected at the pathophysiological level [8]. Therefore, it is potentially important to identify determinants of sleep disorders in middle-aged and older adults as a strategy to prevent cognitive decline and dementia.

Among the many factors studied, diet has been the focus of recent attention due to the potential relation with both sleep quality and its related health outcomes [9]. The Mediterranean dietary pattern has gained popularity over recent decades due to its palatable taste and a strong evidence of benefits for health [10]. Despite a single definition of Mediterranean diet cannot be achieved, it refers to the traditional diet of Southern Italian people explored in the 60s by Ancel Keys, characterized by certain peculiarities including high consumption of plant-based foods (such as fruit, vegetable, legumes and nuts), preference for whole-grain cereals, fish (whenever available) and dairy products instead of other sources of refined carbohydrates and animal proteins, respectively; other characteristics were the daily consumption of olive oil and moderate intake of alcohol (mostly red wine) during meals [11]. A combination of these features has been further investigated in several studies, leading to the development of a number of adherence scores ideally optimized for type of population (i.e., geographical localization), diet parameters availability (i.e., completeness of dietary questionnaires), and generalizability of results (i.e., use of comparable scores) [12]. High adherence to the Mediterranean diet has been associated with a number of cardio-metabolic health outcomes [13,14], including lower risk of cardiovascular-related disorders [15,16], diabetes [17], metabolic syndromes [18,19], and non-alcoholic fatty liver disease [20,21]. These beneficial effects are ascribed to various mechanisms, mostly involving a high content in antioxidants and healthy dietary fats, which in turn may improve insulin sensitivity, reduce vascular inflammation and improve endothelial dysfunction [22]. Lately, a large body of literature has also shown that adherence to a Mediterranean dietary pattern may exert benefits also toward mental health and neurological outcomes, including stroke, cognitive impairment, depression, and dementia [23,24,25,26,27,28]. Recent evidence shows a relation between adherence to the Mediterranean diet and sleep duration and quality in adults [29,30,31], but only few studies have been performed and more research is warranted to better investigate such a relation. The aim of this study was to evaluate the association between sleep quality and adherence to the Mediterranean dietary pattern in a sample of Italian adults.

## 2. Materials and Methods 

### 2.1. Study Population

The Mediterranean healthy Eating, Aging, and Lifestyles (MEAL) study is cross-sectional study aimed to explore the relation between nutritional and lifestyle behaviors characterizing individuals living in the Mediterranean area. The detailed study protocol with the rationale, design, and methods has been described in detail elsewhere [32]. Briefly, the cohort consisted of a random sample of men and women (age 18+ years) registered in the records of local general practitioners in the urban area of Catania, one of the largest cities in the east coast of Sicily, southern Italy, during 2014–2015. The sampling technique included stratification by municipality area, age, and sex of inhabitants, and randomization into subgroups, with randomly selected general practitioners being the sampling units, and individuals registered to them comprising the final sample units. Pregnant women were not considered in this study. Participants randomly selected for recruitment were stratified by sex and 10-year age groups. The theoretical sample size was set at 1500 individuals to provide a specific relative precision of 5% (Type I error, 0.05; Type II error, 0.10), taking into account an anticipated 70% participation rate. Out of 2405 individuals invited, the final sample size was 2044 participants (response rate of 85%). All the study procedures were carried out in accordance with the Declaration of Helsinki (1989) of the World Medical Association. Participants provided written informed consent and the study protocol was approved by the ethics committee of the referent health authority.

### 2.2. Data Collection

Data regarding demographic (i.e., age, sex, educational and occupational level) and lifestyle characteristics (i.e., physical activity, smoking and drinking habits) were collected. Educational level was categorized as: (i) low (primary/secondary), (ii) medium (high school), and (iii) high (university). Occupational level was classified as: (i) unemployed, (ii) low (unskilled workers), (iii) medium (partially skilled workers), and (iv) high (skilled workers). Physical activity level was assessed using International Physical Activity Questionnaires (IPAQs) [33], which are comprised a set of questionnaires (5 domains) on time spent being physically active in the last 7 days that allow categorization of physical activity as: (i) low, (ii) moderate, and (iii) high. Smoking status was classified as: (i) non-smoker, (ii) ex-smoker, and (iii) current smoker. Alcohol consumption was categorized as (i) none, (ii) moderate drinker (0.1-12 g/d) and (iii) regular drinker (>12 g/d). Anthropometric measurements were performed according to standardized methods [34]. Height was measured to the nearest 0.5 cm without shoes, with the back square against the wall tape, eyes looking straight ahead, with a right-angle triangle resting on the scalp and against the wall. Body mass index (BMI) was calculated, and patients were categorized as under/normal weight (BMI <25 kg/m^2^), overweight (BMI 25 to 29.9 kg/m^2^), and obese (BMI ≥30 kg/m^2^) [35].

### 2.3. Dietary Assessment

Dietary data was collected using long and short food frequency questionnaires (FFQs), developed and previously validated for the Sicilian population [36,37]. The FFQs consisted of 110 food and drink items representative of the diet during the previous 6 months. Participants of the study were asked how often, on average, they had consumed foods and drinks included in the FFQ, with nine responses ranging from "never" to "4–5 times per day". Intake of food items characterized by seasonality referred to consumption during the period in which the food was available and then adjusted by its proportional intake over one year. After exclusion of 107 entries with unreliable intakes (<1000 or >6000 kcal/d, controlled case by case and validated due to missing food items or unreliable answers), a total of 1936 individuals were included in the analyses for the present study.

### 2.4. Adherence to the Mediterranean Diet

Mediterranean diet adherence was assessed using the score developed by Sofi et al. [15]. Briefly, a scoring system (the MEDI-LITE score) was built based on existing literature weighting all the median (or mean) values for the sample size of each study population and then calculating a mean value of all the weighted medians; hence, two standard deviations were used to determine three different categories of consumption for each food group. For food groups, typical of the Mediterranean diet (fruit, vegetables, cereals, legumes and fish), two points were given to the highest category of consumption, one point for the middle category and zero points for the lowest category of intake. Contrariwise, for food groups not typical of the Mediterranean diet (meat and meat-based products, dairy products), two points were given for the lowest category, one point for the middle category and zero points for the highest category of consumption. Regarding alcohol, categories related to the alcohol unit (one alcohol unit = 12 g of alcohol) were used by giving two points to the middle category (1–2 alcohol units/d), one point to the lowest category (>1 alcohol unit/d) and zero points to the highest category of consumption (>2 alcohol units/d). The final score comprised nine food categories (including olive oil) with a score ranging from zero points (lowest adherence) to 18 points (highest adherence).

### 2.5. Sleep Quality

The Pittsburg sleep quality index (PSQI) [38] was used to assess participants’ sleep quality and disturbances in the past six month. It consists of 19 items which are rated on a four-point scale (0–3) and grouped into seven components (sleep quality, sleep latency, sleep duration, habitual sleep efficiency, sleep disturbance, use of sleeping medications, and daytime dysfunction). The item scores in each component were summed and converted to component scores ranging from 0 (better) to 3 (worse) based on guidelines. Total PSQI scores were calculated as the summation of seven component scores ranging from zero to 21, where higher score indicates worse condition. A total global PSQI score of <5 is indicative of adequate sleep quality.

### 2.6. Statistical Analysis

Categorical variables are presented as frequencies of occurrence and percentages differences between groups were tested using a Chi-squared test. First, the difference between distribution of background variables by sleep quality (adequate vs. inadequate) was tested. Second, differences in distribution of sleep-related characteristics between groups of individuals divided into quartiles of Mediterranean diet adherence score (Q1 had the lowest adherence, Q4 had the highest adherence) was tested. The relation between adherence to the Mediterranean diet and sleep-related outcomes was tested through multivariate logistic regression analysis adjusted for baseline characteristics (age, sex, marital, educational and occupational status, smoking and alcohol drinking habits, and physical activity level) comparing individuals grouped into quartiles or estimating the association by 1-point increase of the Mediterranean diet adherence score. A sensitivity analysis excluding, one at a time, each individual component of the Mediterranean diet adherence score was performed. Finally, a subgroup analysis by weight status categorization (normal/overweight and obese individuals) has been performed to test stability of results. All reported *p* values were based on two-sided tests and compared to a significance level of 5%. SPSS 17 (SPSS Inc., Chicago, IL, USA) software was used for all the statistical analysis.

## 3. Results 

A total of 1314 individuals (67.9% of the sample) reported an overall adequate sleep quality according to the PSQI score. The distribution of the baseline characteristics of the study participants by sleep quality revealed that there were no significant differences between groups with the exception of occupational level, as there was a significantly higher proportion of individuals with adequate quality of sleep in the highest category than in the lower. However, the distribution was not linear, and a high proportion of individuals with adequate quality of sleep were present also in the lowest category (Table 1).

The relation between specific indicators of sleep quality of the study participants by quartiles of the Mediterranean diet adherence score are reported in Table 2. Among participants more adherent to the dietary pattern (the highest quartile, Q4) there was a higher proportion of individuals with overall better sleep quality compared to the less adherent (the lowest quartile, Q1; 72.4% vs. 58.9%; P <0.001); among specific domains of the PSQI, significantly lower occurrences of shorter sleep durations, longer sleep latency, day dysfunction due to sleepiness, very low sleep efficiency and self-reported sleep quality occurred among participants in the highest quartile of the Mediterranean diet adherence score.

Among the whole sample, a higher adherence to the Mediterranean diet was associated with a higher likelihood of adequate overall sleep quality (highest vs. lowest quartile, OR = 1.82, 95% CI: 1.32, 2.52; Table 3). However, among the specific indicators of sleep quality, only sleep latency was significantly associated with higher adherence to the dietary pattern, while no day dysfunction due to sleepiness was associated with the third quartile of the Mediterranean diet adherence score, but not with the highest. When considering the relation with 1-point increase of the Mediterranean diet adherence score, the multivariate regression analysis revealed that individuals were 10% more likely to have an overall adequate sleep quality, while among individual components of the PSQI score, 1-point increase of the dietary adherence score was significantly associated with having adequate sleep duration, latency, and efficiency (Table 3).

An alternative analysis by excluding one at the time each individual component of the Mediterranean diet adherence score was performed in order to test whether any of these could explain alone the association of the score (Table 4). The results show that the association was robust, as the association with overall sleep quality was significant in all alternative scores; moreover, exclusion of no individual component, besides olive oil, showed significant association with the aforementioned aspects of sleep quality, including sleep duration, latency, and efficacy, suggesting that olive oil may play an independent role in sleep quality.

Table 5 summarizes the results of a supplementary analysis, in which the associations of all endpoints were tested separately according to body weight status of study participants, leading to some differences: Specifically, the association between adequate sleep quality and higher adherence to the Mediterranean diet was observed only among normal/overweight individuals (highest vs. lowest quartile, OR = 2.30, 95% CI: 1.49, 3.54; 1-point increase, OR = 1.10, 95% CI: 1.04, 1.16), while this was not found in obese participants. Among the specific indicators of sleep quality, only sleep latency was associated with the diet score in the former group, but not in the latter (Table 5).

## 4. Discussion

In the present study, a relation between sleep quality and adherence to the Mediterranean dietary pattern has been reported in a cohort of Southern Italian adults. Among the main domains investigated, only sleep latency resulted in being independently associated with higher adherence to this dietary pattern, suggesting that the overall sleep quality rather than specific aspects are associated with a healthier diet. Considering the impact of sleep-related habits toward adverse health outcomes, it is crucial to investigate and identify potential dietary determinants of sleep quality.

To our knowledge, only two studies previously investigated the association between adherence to the Mediterranean diet and sleep parameters in adults [29,30]. One study was conducted on about 1500 older adults living in Spain followed up for 2.8 years and monitored for their sleep duration and indicators of poor sleep quality. The authors found that individuals more adherent to the Mediterranean dietary pattern had a lower risk of a variation (increase or decrease) in sleep duration of more than 2 h and were also at lower risk of poor sleep quality [29]. Another study investigated the relation between adherence to the Mediterranean diet and specific aspects of sleeping, such as insomnia symptoms, finding a positive effect with adherence to a Mediterranean dietary pattern [30]. Some studies investigated the association between sleep duration and overall diet quality [39,40], while others also explored the relation between sleep patterns and eating behaviors, such as unbalanced food variety, irregular meal times, snacking between meals, eating out, and other potentially unhealthy eating habits [41,42]. Concerning our specific findings on sleep latency, intervention studies suggest a causal association between higher fat and carbohydrate intake close to bedtime and high sleep latency [43], thus confirming our results. In general, a consistent relation between dietary behaviors, nutrition quality, and sleep-related habits has been reported in most of the aforementioned studies. However, the direction of the association is debatable, whether better dietary habits might lead to better sleeping patterns or the other way around. In fact, experimental studies demonstrated both ways of association: on one side, it has been demonstrated that a high-quality diet improved sleep duration; on the other, it has been shown that sleep deprivation may increase appetite for high-calorie foods [44].

The Mediterranean dietary pattern may assure an adequate nutritional profile, including high consumption of fruit, vegetable, fish, whole-grains, olive oil, and limited amounts of meat, dairy and alcohol [45]. Previous reports from the cohort investigated in this study showed a significant inverse relation between higher adherence to the Mediterranean diet and likelihood of being obese [46], hypertensive [47] or suffer from dyslipidemia [48]. However, no individual component of the Mediterranean diet has been shown to be responsible alone for such associations, while some evidence on consumption of certain classes of polyphenols (such as flavonoids, phenolic acids and phytoestrogens) may explain, at least in part, these previous findings [49,50]. Similar considerations have been drafted while examining the association between higher adherence to the Mediterranean diet and mental health, which in turn might be associated with improved sleep patterns [51,52]. Richness of the Mediterranean diet in bioactive compounds with beneficial effects, such as antioxidant or anti-inflammatory properties, may exert neuroprotection and reduce oxidative damage and cerebral ischemia [53]. In fact, impaired antioxidant defense responses, such as increased rate of oxidative processes in several organs, including heart, liver and brain, have been reported during sleep deprivation while increased neuro-inflammation has been postulated to contribute to poor sleep quality [54,55]. Further evidence also shows that sleep duration and quality may be mediated by C-reactive protein (CRP), γ-glutamyl transferase (GGT), carotenoids, uric acid, and some vitamins, including vitamin C and D [56,57]. The high content of the Mediterranean diet in polyunsaturated fatty acids (PUFA) and phytochemicals, such as polyphenols, have been demonstrated to have an impact on inflammatory biomarkers [58]. Cohort studies have shown an inverse association between dietary PUFA [59,60] and polyphenols with better mental health (i.e., depressive symptoms, cognitive impairment, etc.) [61,62,63]. A variety of neuroprotective activities have been described, including anti-amyloidogenic efficacy, neuroprotection via modulation of neural mediators, and modulation of different signaling pathways [64,65]. Moreover, environmental stimuli (including exercise, but also sleep and dietary patterns) have been linked to hippocampal neurogenesis, a phenomenon occurring also in human adults, that seems to be linked to a number of pathological conditions, including stress, anxiety and depression, and cognitive impairment [66]. The resulting benefits of high adherence to the Mediterranean diet on sleep, cognition, mood, and Alzheimer’s disease may, thus, also depend on the enhancement of structural and functional brain plasticity mediated by components of this dietary pattern, such as PUFA and polyphenols [67,68].

In addition to the aforementioned potential mechanisms, in this study we also hypothesized that the association between adherence to the Mediterranean diet and sleep quality might somehow mediate the effects of obesity on sleep quality; this relation has been reported in previous papers [69], but rarely investigated in light of dietary factors associated with weight status. In a sub-analysis of the present study we found that adherence to the Mediterranean diet was significant in normal and overweight individuals, but was not evident in the obese. Prospective cohort studies showed evidence of a causal relation between short sleep duration and occurrence of obesity at later age [70]. The most studied mechanism relating sleep and body weight regards the balance between leptin and ghrelin, two hormones involved in food intake and energy balance which have been demonstrated to be altered following sleep disturbances [71]. Leptin is an adipocyte-derived hormone that suppresses hunger and stimulates energy expenditure while ghrelin is stomach-derived peptide that stimulates appetite and fat production. Some studies showed that short sleep and sleep deprivation may decrease circulating leptin and increase ghrelin levels [72], despite findings not being univocal [73,74]. Among other hormones potentially involved in the relation between sleep quality and body weight, some studies showed that sleep disturbances may increase morning cortisol levels, inhibit insulin sensitivity and growth hormone secretion [75,76]. The relation between poor sleep and obesity has been widely demonstrated, and also the other way around, where excess body weight may favor the occurrence of sleep apnea, which in turn causes scarce sleep quality [77]. Most important, recent evidence shows that obstructive sleep apnea may have an impact on the structure and function of blood vessels, adversely affecting cognition in addition to culminating in mortality and morbidity [78]. Hypoxia, hypertension, hypo-perfusion, endothelial dysfunction, inflammation, and oxidative stress noted in obstructive sleep apnea patients also occur in Alzheimer’s disease patients, suggesting a pathological commonality that may relate both conditions [79]. In this context, higher adherence to a Mediterranean dietary pattern has been proven to provide advantages on metabolic profiles and long-term weight status maintenance [80,81]. Also in this regard, Mediterranean dietary polyphenols have been hypothesized to potentially play a role in weight management through a number of mechanisms, including activation of β-oxidation; a prebiotic effect for gut microbiota; induction of satiety; stimulation of energy expenditure by inducing thermogenesis in brown adipose tissue; modulation of adipose tissue inhibiting adipocyte differentiation; promotion of adipocyte apoptosis and increasing lipolysis [82,83]. Thus, it may be possible that the association between adherence to the Mediterranean diet and sleep quality retrieved in our study may, in fact, be mediated by a better weight status. This hypothesis will need further exploration in future studies.

The findings of this study should be considered in light of some limitations. First, the real direction of the associations retrieved cannot be identified through cross-section studies and reverse causation should be taken into account as potential explanation of the results presented. It is noteworthy to emphasize that even with a prospective study design, the possibility that sleep and dietary patterns are part of an overall healthier or unhealthier lifestyle pattern cannot be ruled out, and that only further research into mechanistic and experimental studies would clarify the nature of the association. Second, the use of self-reported FFQs and sleep quality tools may be affected by recall and social desirability biases. However, the tools used in this study are well-established instruments to investigate the research question proposed and methods are comparable to the existing literature. Third, given the variety of Mediterranean adherence scores used in the literature, results may not be directly comparable with studies using other instruments. However, the adherence score used in the present study is based on the summary of scientific literature providing evidence of association between the Mediterranean diet and health outcomes, suggesting the robustness of the instrument. Forth, despite controlling for occupational status, we were unable to test the role of financial allowance in the study participants, which might play a role in adherence to the Mediterranean diet and could be further investigated. Moreover, within the same category of occupational status we had no data for jobs that possibly required night shifts or had characteristics that might have influenced sleeping patterns. However, assuming a random distribution for such types of jobs (meaning not associated with adherence to the Mediterranean diet), this potential bias should be non-differential among exposure groups.

## 5. Conclusions

In conclusion, high adherence to a Mediterranean dietary pattern is associated with better sleep quality, either toward a direct effect on health or indirect effects through improvement of weight status. Further research should explore whether investigating sleep quality within the context of adherence to the Mediterranean diet might be part of an overall healthier lifestyle pattern, and should investigate the topic with a prospective and longitudinal study design. Future experimental studies are needed to test the impact of sleep quality on health and dietary intake allowing to investigate on causality and mechanisms. Finally, the potential mediating effect of weight status on the relation between Mediterranean diet and sleep quality requires further investigation.

## Figures and Tables

**Table 1:** Baseline characteristics of the study participants by sleep quality. *n* indicates the number of individuals that satisfy each condition within the total sample; % indicates the percentages of individuals that satisfy each condition within the total sample.

	Sleep Quality		
	Inadequate (*n* = 622)	Adequate (*n* = 1314)	*p*-Value
**Sex, *n* (%)**			0.052
*Men*	278 (44.7)	526 (40.0)	
*Women*	344 (55.3)	788 (60.0)	
**Age groups, *n* (%)**			0.161
*<30*	124 (19.9)	226 (17.2)	
*30–49*	218 (35.0)	485 (36.9)	
*50–69*	209 (33.6)	416 (31.7)	
*≥70*	71 (11.4)	187 (14.2)	
**Educational status, *n* (%)**			0.119
*Low*	224 (36.0)	473 (36.0)	
*Medium*	248 (39.9)	472 (35.9)	
*High*	150 (24.1)	369 (28.1)	
**Occupational status, *n* (%)**			0.011
*Unemployed*	131 (24.8)	330 (29.2)	
*Low*	84 (15.9)	181 (16.1)	
*Medium*	167 (31.6)	273 (24.2)	
*High*	146 (27.7)	345 (30.5)	
**Smoking status, *n* (%)**			0.595
Never smoker	375 (60.3)	820 (62.4)	
Former smoker	89 (14.3)	187 (14.2)	
Current smoker	158 (25.4)	307 (23.4)	
**Physical activity level, *n* (%)**			0.169
*Low*	93 (16.6)	236 (20.2)	
*Moderate*	291 (52.0)	565 (48.4)	
*High*	176 (31.4)	367 (31.4)	
**Health status, *n* (%)**			
*Hypertension*	292 (46.9)	684 (52.1)	0.036
*Type-2 diabetes*	45 (7.2)	101 (7.7)	0.725
*Dyslipidemias*	118 (19.0)	238 (18.1)	0.649
*Cardiovascular disease*	57 (9.3)	97 (7.6)	0.198
*Cancer*	19 (3.1)	59 (4.5)	0.134
**Weight status, *n* (%)**			0.372
*Normal*	267 (47.6)	584 (47.2)	
*Overweight*	205 (36.5)	425 (34.4)	
*Obese*	89 (15.9)	228 (18.4)	

**Table 2:** Overall sleep quality and sleep-related characteristics of the study participants by quartiles of Mediterranean diet adherence score. *n* indicates the number of individuals that satisfy each condition within the total sample; % indicates the percentages of individuals that satisfy each condition within the total sample.

	Mediterranean Diet Adherence Score *				
	Q1	Q2	Q3	Q4	*p*-Value
**Overall sleep quality, *n* (%)**					<0.001
*Adequate*	272 (58.9)	403 (68.0)	440 (72.6)	199 (72.4)	
*Inadequate*	190 (41.1)	190 (32.0)	166 (27.4)	76 (27.6)	
**Sleep duration, *n* (%)**					<0.001
*>7 h*	246 (53.2)	371 (62.6)	376 (62.0)	171 (62.2)	
*6–7 h*	111 (24.0)	130 (21.9)	137 (22.6)	57 (20.7)	
*5–6 h*	65 (14.1)	58 (9.8)	74 (12.2)	33 (12.0)	
*<5 h*	40 (8.7)	34 (5.7)	19 (3.1)	14 (5.1)	
**Sleep disturbance, *n* (%)**					0.311
*None*	53 (11.5)	54 (9.1)	74 (12.2)	35 (12.7)	
*Low*	335 (72.5)	444 (74.9)	451 (74.4)	207 (75.3)	
*Medium*	74 (16.0)	95 (16.0)	81 (13.4)	33 (12.0)	
*High*	0	0	0	0	
**Sleep latency, *n* (%)**					0.003
*Very short*	172 (37.2)	253 (42.7)	298 (49.2)	135 (49.1)	
*Short*	153 (33.1)	210 (35.4)	181 (29.9)	85 (30.9)	
*Medium*	101 (21.9)	94 (15.9)	97 (16.0)	41 (14.9)	
*Long*	36 (7.8)	36 (6.1)	30 (5.0)	14 (5.1)	
**Day dysfunction, *n* (%)**					<0.001
*None*	296 (64.1)	433 (73.0)	440 (72.6)	201 (73.1)	
*Low*	75 (16.2)	91 (15.3)	93 (15.3)	30 (10.9)	
*Medium*	35 (7.6)	28 (4.7)	27 (4.5)	23 (8.4)	
*High*	56 (12.1)	41 (6.9)	46 (7.6)	21 (7.6)	
**Sleep efficiency, *n* (%)**					<0.001
*High*	296 (64.1)	433 (73.0)	440 (72.6)	201 (73.1)	
*Medium*	75 (16.2)	91 (15.3)	93 (15.3)	30 (10.9)	
*Low*	35 (7.6)	28 (4.7)	27 (4.5)	23 (8.4)	
*Very low*	56 (12.1)	41 (6.9)	46 (7.6)	21 (7.6)	
**Self-rated sleep quality, *n* (%)**					0.043
*Very low*	14 (3.0)	29 (4.9)	15 (2.5)	13 (4.7)	
*Low*	17 (3.7)	15 (2.5)	15 (2.5)	0 (0)	
*Medium*	17 (3.7)	26 (4.4)	20 (3.3)	11 (4.0)	
*High*	414 (89.6)	523 (88.2)	556 (91.7)	251 (91.3)	
**Need medication to sleep, *n* (%)**	48 (10.4)	70 (11.8)	50 (8.3)	24 (8.7)	0.187

* Groups represent individuals divided into quartiles.

**Table 3:** Association between overall and individual domains of sleep quality and adherence and quartiles of the Mediterranean diet adherence score. Odds ratios indicate the probability that a subject was an adequate sleeper to the probability that the subject was not, between subjects included in each quartile, compared to those included in the lowest.

	Mediterranean Diet Adherence Score				
	Q1	Q2	Q3	Q4	1-Point Increment
	OR (95% CI) *				
**Adequate sleep quality**	1	1.48 (1.15, 1.90) ^§^	1.85 (1.43, 2.39) ^§^	1.82 (1.32, 2.52) ^§^	1.10 (1.05, 1.16) ^§^
**Sleep duration**	1	1.39 (1.04, 1.86) ^§^	1.29 (0.97, 1.71)	1.35 (0.94, 1.92)	1.07 (1.02, 1.12) ^§^
**Sleep disturbance**	1	0.81 (0.51, 1.30)	1.26 (0.82, 1.95)	1.31 (0.77, 2.21)	1.04 (0.97, 1.12)
**Sleep latency**	1	1.12 (0.84, 1.50)	1.64 (1.23, 2.17) ^§^	1.52 (1.07, 2.16)	1.07 (1.02, 1.12) ^§^
**Day dysfunction**	1	1.12 (0.85, 1.49)	1.42 (1.07, 1.88) #	1.25 (0.88, 1.77)	1.04 (1.00, 1.09) #
**Sleep efficiency**	1	1.36 (1.00, 1.84) #	1.33 (0.98, 1.80)	1.40 (0.95, 2.05)	1.06 (1.01, 1.12) #
**Need medication to sleep**	1	0.67 (0.42, 1.07)	1.34 (0.80, 2.25)	1.05 (0.57, 1.93)	1.03 (0.95, 1.11)
**Self-rated sleep quality**	1	1.04 (0.73, 1.48)	1.16 (0.83, 1.64)	1.30 (0.86, 1.98)	1.04 (0.98, 1.09)

* adjusted for age (continuous), sex (male/female), BMI (<25 kg/m^2^, 25–30 kg/m^2^, >30 kg/m^2^), physical activity (low/medium/high), educational status (low/medium/high), occupational status (unemployed/low/medium/high), smoking status (current/former/never), alcohol consumption (no/moderate/regular), health status (presence of hypertension, type-2 diabetes, dyslipidaemias, cardiovascular disease, cancer), and total energy intake. # indicates *p* < 0.05. § indicates *p* < 0.001.

**Table 4:** Association between overall and individual domains of sleep quality and alternative Mediterranean diet adherence scores with exclusion of each individual component one at the time. Odds ratios indicate the probability that a subject was an adequate sleeper to the probability that the subject was not, between subjects included in each 1-point score, compared to those having 1 unit lower.

	Mediterranean Diet Adherence Score, 1-Point Increment Recalculated Excluding:								
	Fruit	Vegetable	Legume	Dairy	Whole-grain	Fish	Meat	Olive oil	Alcohol
	OR (95% CI) *								
**Overall sleep quality**	1.12 (1.06, 1.18) ^§^	1.11 (1.06, 1.17) ^§^	1.10 (1.05, 1.17) ^§^	1.13 (1.07, 1.19) ^§^	1.09 (1.04, 1.15) #	1.11 (1.06, 1.17) ^§^	1.10 (1.05, 1.15) ^§^	1.09 (1.03, 1.14) #	1.10 (1.05, 1.15) ^§^
**Sleep duration**	1.08 (1.02, 1.14) #	1.07 (1.02, 1.12) #	1.08 (1.03, 1.14) #	1.08 (1.03, 1.14) #	1.06 (1.01, 1.11) #	1.07 (1.02, 1.13) #	1.07 (1.02, 1.13) #	1.04 (0.99, 1.09)	1.06 (1.01, 1.11) #
**Sleep disturbance**	1.03 (0.95, 1.11)	1.03 (0.95, 1.11)	1.06 (0.98, 1.15)	1.07 (0.99, 1.16)	1.05 (0.97, 1.13)	1.06 (0.98, 1.14)	1.03 (0.96, 1.11)	1.03 (0.96, 1.11)	1.04 (0.97, 1.12)
**Sleep latency**	1.08 (1.03, 1.14) #	1.09 (1.03, 1.14) #	1.07 (1.02, 1.13) #	1.07 (1.02, 1.12) #	1.06 (1.01, 1.11) #	1.08 (1.03, 1.14) #	1.08 (1.03, 1.13) #	1.06 (1.01, 1.11) #	1.07 (1.02, 1.12) #
**Day dysfunction**	1.04 (0.99, 1.09)	1.04 (0.99, 1.10)	1.04 (0.99, 1.10)	1.06 (1.01, 1.11) #	1.05 (1.00, 1.10) #	1.05 (1.00, 1.10) #	1.05 (1.00, 1.10)#	1.04 (0.99, 1.09)	1.04 (0.99, 1.09)
**Sleep efficiency**	1.06 (1.00, 1.12) #	1.07 (1.01, 1.12) #	1.06 (1.01, 1.12) #	1.09 (1.03, 1.15) #	1.06 (1.01, 1.11) #	1.06 (1.01, 1.12) #	1.06 (1.01, 1.12) #	1.05 (1.00, 1.11) #	1.07 (1.02, 1.12) #
**Need medication to sleep**	1.04 (0.96, 1.14)	1.03 (0.95, 1.12)	1.03 (0.94, 1.12)	1.02 (0.94, 1.10)	1.03 (0.96, 1.12)	1.04 (0.96, 1.13)	1.04 (0.96, 1.12)	1.02 (0.94, 1.10)	1.02 (0.95, 1.10)
**Self-rated sleep quality**	1.03 (0.96, 1.09)	1.04 (0.98, 1.10)	1.04 (0.98, 1.11)	1.04 (0.98, 1.11)	1.05 (0.99, 1.11)	1.04 (0.98, 1.10)	1.04 (0.98, 1.10)	1.03 (0.97, 1.10)	1.04 (0.98, 1.10)

* adjusted for age (continuous), sex (male/female), BMI (<25 kg/m^2^, 25–30 kg/m^2^, >30 kg/m^2^), physical activity (low/medium/high), educational status (low/medium/high), occupational status (unemployed/low/medium/high), smoking status (current/former/never), alcohol consumption (no/moderate/regular), health status (presence of hypertension, type-2 diabetes, dyslipidaemias, cardiovascular disease, cancer), and total energy intake. # indicates *p* < 0.05. § indicates *p* < 0.001.

**Table 5:** Association between overall and individual domains of sleep quality and adherence and quartiles of the Mediterranean diet adherence score by weight status. Odds ratios indicate the probability that a subject was an adequate sleeper to the probability that the subject was not, between subjects included in each 1-point score, compared to those having 1 unit lower.

	Mediterranean Diet Adherence Score				
	Q1	Q2	Q3	Q4	1-Point Increment
	OR (95% CI) *				
*Normal/overweight*					
Overall sleep quality	1	1.22 (0.87, 1.71)	1.79 (1.27, 2.54) ^§^	2.30 (1.49, 3.54) ^§^	1.10 (1.04, 1.16) ^§^
Sleep duration	1	1.32 (0.94, 1.83)	1.29 (0.92, 1.79)	1.32 (0.89, 1.97)	1.06 (1.01, 1.12) #
Sleep disturbance	1	0.82 (0.47, 1.40)	1.28 (0.77, 2.13)	1.22 (0.67, 2.22)	1.03 (0.95, 1.12)
Sleep latency	1	1.08 (0.77, 1.51)	1.96 (1.40, 2.74) ^§^	1.62 (1.09, 2.41) ^§^	1.09 (1.03, 1.15) #
Day dysfunction	1	1.26 (0.91, 1.74)	1.51 (1.09, 2.10) ^§^	1.27 (0.86, 1.88)	1.03 (0.97, 1.08)
Sleep efficiency	1	1.32 (0.92, 1.88)	1.29 (0.90, 1.84)	1.48 (0.96, 2.28)	1.04 (0.99, 1.10)
Need medication to sleep	1	0.62 (0.37, 1.04)	1.24 (0.69, 2.23)	0.97 (0.50, 1.89)	1.01 (0.92, 1.10)
Self-rated sleep quality	1	0.97 (0.65, 1.46)	1.27 (0.85, 1.89)	1.31 (0.82, 2.08)	1.04 (0.98, 1.11)
*Obese*					
Overall sleep quality	1	0.91 (0.39, 2.15)	1.11 (0.49, 2.50)	1.12 (0.33, 3.79)	1.12 (0.95, 1.32)
Sleep duration	1	1.67 (0.75, 3.73)	1.58 (0.75, 3.36)	2.68 (0.80, 8.96)	1.09 (0.94, 1.26)
Sleep disturbance	1	1.38 (0.42, 4.54)	1.71 (0.59, 4.92)	3.41 (0.81, 14.36)	1.20 (0.98, 1.49)
Sleep latency	1	1.08 (0.49, 2.36)	0.69 (0.33, 1.43)	0.65 (0.21, 2.01)	0.89 (0.77, 1.03)
Day dysfunction	1	0.69 (0.32, 1.49)	1.06 (0.52, 2.19)	1.22 (0.39, 3.79)	1.07 (0.93, 1.23)
Sleep efficiency	1	1.91 (0.81, 4.50)	1.32 (0.61, 2.87)	1.46 (0.44, 4.81)	1.13 (0.97, 1.33)
Need medication to sleep	1	0.48 (0.09, 2.40)	1.36 (0.24, 7.59)	1.57 (0.40, 5.92)	1.21 (0.87, 1.69)
Self-rated sleep quality	1	1.28 (0.54, 3.00)	0.87 (0.38, 1.97)	0.92 (0.25, 3.30)	0.95 (0.81, 1.11)

* adjusted for age (continuous), sex (male/female), BMI (<25 kg/m^2^, 25–30 kg/m^2^, >30 kg/m^2^), physical activity (low/medium/high), educational status (low/medium/high), occupational status (unemployed/low/medium/high), smoking status (current/former/never), alcohol consumption (no/moderate/regular), health status (presence of hypertension, type-2 diabetes, dyslipidaemia, cardiovascular disease, cancer), and total energy intake. # indicates *p* < 0.05. § indicates *p* < 0.001.
